# A Fatty Acid Metabolism Signature Associated With Clinical Therapy in Clear Cell Renal Cell Carcinoma

**DOI:** 10.3389/fgene.2022.894736

**Published:** 2022-07-08

**Authors:** Zhihao Wei, Gong Cheng, Yuzhong Ye, Changjie Le, Qi Miao, Jiawei Chen, Hongmei Yang, Xiaoping Zhang

**Affiliations:** ^1^ Department of Urology, Union Hospital, Tongji Medical College, Huazhong University of Science and Technology, Wuhan, China; ^2^ Institute of Urology, Union Hospital, Tongji Medical College, Huazhong University of Science and Technology, Wuhan, China; ^3^ Department of Pathogenic Biology, School of Basic Medicine, Huazhong University of Science and Technology, Wuhan, China

**Keywords:** lipid metabolism, immunotherapy, sunitinib-resistant, clear cell renal cell carcinoma, risk model

## Abstract

Renal cell carcinoma is one of the most common tumors in the urinary system, among which clear cell renal cell carcinoma is the most common subtype with poor prognosis. As one of the tumors closely related to lipid metabolism, the role of fatty acid metabolism in ccRCC was investigated to predict the prognosis and guide treatment strategies. RNA-seq and clinical information of patients with ccRCC and expression microarray of human renal cell carcinoma cell lines were obtained from TCGA and GEO databases. Fatty acid metabolism–related risk signature was established by the univariate Cox regression and LASSO analysis to predict patient prognosis and response to different treatment modalities. Using the fatty acid metabolism risk signature, the risk score for each sample in the TCGA cohort was calculated and divided into high-risk and low-risk groups, with the cutoff point being the median. Patients with higher risk scores had a poorer prognosis than those with lower risk scores. The response of each sample to immunotherapy was predicted from the “TIDE” algorithm, while the sensitivity of each sample to sunitinib was obtained using the “pRRophetic” R package. Patients with lower risk scores had higher expression of PD-L1 and better efficacy for sunitinib than those in the high-risk group and were less likely to develop drug resistance, while patients with high-risk scores had a strong response to the anti-CTLA4 antibody therapy. A nomogram was constructed by independent prognostic factors to predict the 1-, 3-, and 5-year survival. According to the calibration curves, the nomogram had an excellent ability to predict survival for patients with ccRCC. Therefore, the fatty acid metabolism risk signature we established can not only predict the survival of patients with ccRCC but also predict patient response to targeted therapy and immunotherapy to provide optimal treatment strategies for patients.

## Introduction

Renal cell carcinoma (RCC) is one of the most common malignancies in the urinary system and accounts for >90% of cancers in the kidney (Siegel et al., 2019). Clear cell renal cell carcinoma is the most common subtype and accounts for most cancer-related deaths (Hsieh et al., 2017). Localized ccRCC can be treated with partial or radical surgical resection, but more than 30% of patients will eventually develop metastasis after surgery, which requires systemic therapies including immunotherapy, targeted therapy, and chemotherapy (Nerich et al., 2014). However, resistance to drugs such as sunitinib has undermined the effectiveness of targeted therapy (Joosten et al., 2015).

Metabolic reprogramming is a hallmark of malignancy. Increased lipid uptake, storage, and lipogenesis occur in RCC and contribute to rapid tumor growth (Faubert et al., 2020). Lipids are widely distributed in cellular organelles and are critical components of all membranes. Abnormal *de novo* fatty acids (FAs) and cholesterol biosynthesis supply the membrane and energy substrates for rapidly growing tumor cells and continuously adapt to a variety of microenvironmental conditions conducive to tumor growth (Cheng et al., 2018; Bacci et al., 2021). In most tumors, the process of lipogenesis is upregulated for the energy needs of tumor cells (Guo et al., 2013). Moreover, lipid uptake and storage are also upregulated in most tumors (Zhao et al., 2017). Fatty acids can be used as substrates to generate large amounts of ATP through *β* oxidation processes, thereby energizing tumor cells (Betz and Enerbäck, 2018). Intriguingly, recent studies have found that lipid autophagy in renal carcinoma cells can effectively inhibit tumor progression (Xiao et al., 2019). In summary, the lipid metabolic process in renal carcinoma cells is closely related to tumorigenesis and tumor progression, and the specific mechanism of this process remains unclear.

In recent years, an increasing number of studies have found that lipid metabolism also plays a key and complex role in resistance to antitumor therapy (Cao, 2019). Lipid anabolic rewiring supports disease relapse and drug resistance. Multiple signaling pathways and multiple metabolites of lipid metabolism can all affect the efficacy of tumor therapy and eventually lead to emergence of drug resistance by altering the tumor microenvironment (Bacci et al., 2021). For example, it has been shown that the metabolism of long-chain FA sapienate is enhanced in a variety of solid tumors, accompanied by upregulation of lipid-modifying enzymes, which allows the insertion of a double bond into the acyl chain of FA, thereby bypassing potential antitumor therapy (Peck and Schulze, 2016).The reprogramming of lipid metabolism and changes in the tumor microenvironment not only play a key role in tumor progression but also affect the therapeutic effect of tumors.

In recent years, there is growing evidence of a close relationship between immune cell responses and metabolic reprogramming (Lee et al., 2018). Evidence has shown that some tumor cells increase the uptake of fatty acids, which is directly correlated with CD8^+^ T cell suppression. In addition, the researchers also observed that the combined treatment of the inhibitor of fatty acid transporter 2 (FATP2), lipofermata, with ICIs (anti-CTLA4 antibody), can effectively inhibit tumor progression (Veglia et al., 2019). This suggests that tumor microenvironmental conditions, cellular metabolic reprogramming, and infiltrating immune cells all have overlapping effects and influence the response to treatment (Bleve et al., 2020). The emerging research field of immunometabolism also brings new prospects for immunotherapy in the treatment of tumors.

Lipid metabolism, especially fatty acid metabolism, has an impact on tumor development, progression, drug resistance, and immunotherapy, so we wonder if the characteristics of lipid metabolism pathways can be used to predict the prognosis, responsiveness to TKI (such as sunitinib), and sensitivity to immunotherapy in patients with renal cancer. Also, the fatty acid metabolism–related gene set in ccRCC has not been systematically studied. Herein, we downloaded the transcriptome profiling and clinical data of 538 clear cell renal cell carcinoma samples and 72 normal kidney tissue samples from the Cancer Genome Atlas (TCGA) portal. We screen differentially expressed genes in three fatty acid metabolism pathways and identified a prognostic signature by using the univariate Cox regression analysis and LASSO–penalized Cox analysis. The prognostic signature independently predicted the overall survival and sensitivity to sunitinib. Moreover, the signature could also predict patient response to immunotherapy. These studies provide a new perspective for prognostic prediction of ccRCC patients and guide our choice of treatment methods through the level of risk scores.

## Methods and Materials

### Data Acquisition and Processing

The RNA-seq expression files and relevant clinicopathological and survival data of TCGA-KIRC patients were acquired from the TCGA database, including 538 ccRCC and 72 normal kidney tissue samples. All the transcriptome profiling included both HTSeq-Counts and HTSeq-FPKM workflow types, and we calculated the corresponding TPM using the following formula: $TPM_{i}=\left(\frac{FPKM_{i}}{\sum_{i}FPKM_{j}}\right)\times 10^{6}$ . Moreover, the microarray data profiles of GEO: GSE183140 and GSE150404 were also downloaded from the GEO database, the former consisting of 18 sunitinib-resistant renal cell lines and nine originator cell lines and the latter including 60 expression data of clear cell renal cell carcinoma samples at different pathological stages (Supplementary Table S1). Similarly, clinical information of each sample of GSE150404 was downloaded from the GEO database.

### Differentially Expressed Genes

The R package “edgeR” was used to identify differentially expressed genes between 538 ccRCC samples and 72 normal tissue samples of the TCGA cohort, and the cutoff point was defined as |logFC| > 1 and *p* < 0.05.

### Establishment of a Fatty Acid Metabolism Risk Signature

A total of 75 fatty acid metabolism–related genes were obtained from the website (https://www.genome.jp/kegg/), and 23 genes were included in the list of differentially expressed genes. Univariate Cox regression analyses of these 23 genes were performed to construct the risk model, and 10 genes met the condition that *p* < 0.05. By using the “glmnet” R package, we further screened out four genes credibly associated with the prognosis of ccRCC patients to establish this risk model by the LASSO–penalized Cox analysis. Coefficients of these four genes (ACADM, ACAT1, CPT1B, and HACD1) were used to calculate the risk score of each sample as shown in the following:

$$\begin{array}{l} Risk\;score\;=\left(-0.0164225144171802\right)\;\times ACADM\\ +\left(-0.0062944739645312\right)\times ACAT1\\ +\left(0.338329366345878\right)\times CPT1B\\ +\left(0.45060041098045\right)\times HACD1 \end{array}$$ (1)

Samples from the TCGA cohort and the GSE 150404 were divided into a high-risk group and a low-risk group by the median risk scores.

### The Functional Enrichment Analysis

In the same way, as aforementioned, we screened for differentially expressed genes between the high-risk group and low-risk group. Using the “clusterProfiler” R package, we performed the GO and KEGG functional enrichment analyses based on these differentially expressed genes. In addition, the REACTOME functional enrichment analysis was also performed by the “ReactomePA” R package. The significant pathways were determined by a cutoff value of *p* value < 0.05.

### The Single-Sample Gene Set Variation Analysis

“c2.cp.v7.5.1.symbols.gmt” was obtained as the reference gene sets by using the “GSEABase” R package. The single-sample gene set variation analysis of each sample in the high-risk group and low-risk group was conducted by the “GSVA” R package. Thus, differential pathways between the high-risk group and low-risk group were obtained. FDR <0.05 indicated a statistically significant pathway. Finally, five pathways related to fatty acid metabolism were selected for further analysis.

### Comparison of Somatic Mutation Profiles

The mutation landscape of 336 tumor samples from the “TCGA-KIRC” project was evaluated using the “maftools” R package. Meanwhile, mutation landscapes of 140 tumor samples in the high-risk group and 184 samples in the low-risk group were obtained. The tumor mutation burden (TMB) was measured according to tumor-specific mutated genes (Budczies et al., 2019).

### Establishment of a Nomogram

The univariate Cox regression and multivariate Cox regression analysis were performed to screen the indicators for OS prediction. By using the “rms” R package, a nomogram with the independent indicators such as age, pathological grade, pathological stage, the AJCC M stage, and prognostic risk score model was established for predicting OS in ccRCC. In order to verify the predictive validity of the nomogram for OS, calibration curves for 1-, 3-, and 5-year OS were constructed.

### Survival Analysis

Differences between the high-risk group and low-risk group in OS were represented by the Kaplan–Meier curve using the “survival” and “surviminer” R packages.

### Predicting Immune Cell Infiltration and Response to Immunotherapy

“Cell Type Identification by Estimating Relative Subsets of RNA Transcripts (CIBERSORT)” is an analytical tool developed by Newman to provide an estimate of the abundance ratio of member cell types in a mixed cell population using gene expression data (Newman et al., 2015). The leukocyte signature matrix (LM 22) was downloaded from the website (https://cibersort.stanford.edu), which contained 547 genes that distinguished 22 human hematopoietic cell phenotypes. Next, we calculated the abundance ratio matrix of 22 immune cells of each sample in the high-risk group and low-risk group and compared whether the abundance of infiltration of 22 immune cells in the two groups was significantly different.

The response to immunotherapy was mainly reflected in the response to PD-L1 and CTLA4 inhibitors in our case. The Tumor Immune Dysfunction and Exclusion (TIDE) (http://tide.dfci.harvard.edu) algorithm could estimate multiple published transcriptomic biomarkers to predict patient response (Jiang et al., 2018). The response to immunotherapy of each sample in the TCGA cohort was evaluated by the TIDE module.

The “pRRophetic” R package was used to predict the response to the targeted drug sunitinib of each sample of the TCGA cohort.

### Clinical Patient Samples

The seven paired clinical samples were all obtained from ccRCC patients who underwent surgical treatment at the Department of Urology, Union Hospital Affiliated to Tongji Medical College (Wuhan, China) from 2016 to 2021. They had never received preoperative chemotherapy or radiotherapy and informed consent was signed. After the tumor tissues and matched para-carcinoma renal tissues were isolated, both of them were frozen in liquid nitrogen quickly to prevent total RNA and protein degradation. The study was approved by the Human Research Ethics Committee of Huazhong University of Science and Technology. The study complies with the guidelines of the Declaration of Helsinki.

### Cell Culture and Establishment of Sunitinib-Resistant Cell Lines

The two kinds of human renal cell carcinoma cell lines: 786-O and CAKI-1 were used in this study and were purchased from the American Type Culture Collection. The cells were grown in Dulbecco’s modified Eagle’s medium containing high glucose (4.5 g/L), fetal bovine serum (10%), and penicillin/streptomycin solution (1%). All cells were cultured under standard conditions: at 37°C in a 5% CO_2_ atmosphere.

Sunitinib was purchased from MedChemExpress (Shanghai, China), and the stock solutions (10 mmol/L) were prepared using DMSO and stored at −20°C. 786-O and Caki-1 cells were treated with sunitinib at an initial concentration of 2.5 and 5 μm, respectively, and the concentration of sunitinib was increased stepwise by 0.5 μm to gradually establish sunitinib-resistant 786-O (10 μm sunitinib) and Caki-1 (5 μm sunitinib) cells.

### RNA Extraction and qRT-PCR

The clinical tissue specimens were prepared by grinding, while cells were collected before mixing with the TRIzol reagent (Thermo Fisher Scientific; Waltham, MA, United States) to isolate and extract total RNA from tissues and cells. A reverse transcription method was conducted to amplify the corresponding mRNA we need. All the qPCR analyses were performed using the Step One Plus (ABI; Thermo Fisher Scientific, Rockford, IL, United States) platform using the SYBR Green mix (Thermo Fisher, Massachusetts, United States). Primers of ACADM, ACAT1, CPT1B, HACD1, and GAPDH were designed and purchased from TsingKe (Wuhan, China) and could be traced in Table 1.

**TABLE 1:** Primers of ACADM, ACAT1, CPT1B, HACD1, and GAPDH.

Genes	F 5′-3′	R 5′-3′
ACADM	ACA​GGG​GTT​CAG​ACT​GCT​ATT	TCC​TCC​GTT​GGT​TAT​CCA​CAT
ACAT1	TAC​CAG​AAG​TAA​AGC​AGC​ATG​G	TCA​TTC​AGT​GTA​CTG​GCA​TTG​G
CPT1B	GCG​CCC​CTT​GTT​GGA​TGA​T	CCA​CCA​TGA​CTT​GAG​CAC​CAG
HACD1	GGT​GTG​GCT​CAT​TAC​TCA​CAG	GGT​CAA​GAA​GGC​TGA​ATG​TGT
GAPDH	GGA​GCG​AGA​TCC​CTC​CAA​AAT	GGC​TGT​TGT​CAT​ACT​TCT​CAT​GG

### Statistical Analysis

The Wilcoxon rank-sum test was used to compare the difference between the two groups. The K-W test was performed to compare three or more groups. A chi-square test was performed for comparison of categorical data, while Student’s t-tests were used for continuous data. Statistical significance was defined as *p* < 0.05. All statistical analyses were conducted using R 4.1.2.

## Results

### Identification of Fatty Acid Pathway Risk Signature

**FIGURE 1:**
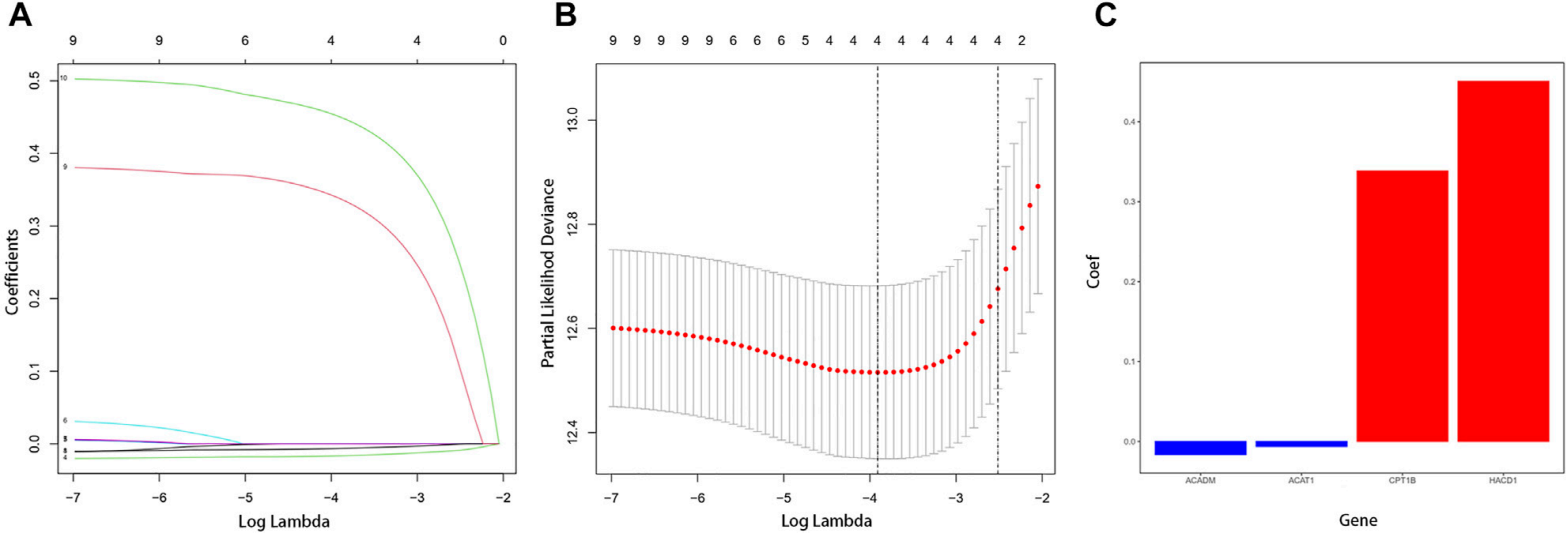
Establishment of fatty acid metabolism risk signature. **(A)** LASSO coefficient profiles for the 10 differentially expressed genes. **(B)** Optimal parameter (λ) was chosen by cross-validation. **(C)** Coefficient values of the four fatty acid metabolism–related genes screened by the LASSO analysis.

A total of 9447 differentially expressed genes were obtained by comparing gene expression between 538 ccRCC samples and 72 normal tissue samples of the TCGA cohort. The differentially expressed genes were analyzed with the “edgeR” R package and under the condition that |logFC| > 1 and *p* < 0.05. Meanwhile, a total of 75 fatty acid metabolism–related genes of three fatty acid pathways (hsa00061: fatty acid biosynthesis, hsa00062: fatty acid elongation, and hsa00071: fatty acid degradation) were investigated, 23 of which were included in the aforementioned differentially expressed genes (Supplementary Table S2). Ten of these genes were significantly correlated with OS in the univariate Cox regression analysis (*p* < 0.05). With the “glmnet” R package, the Lasso-penalized Cox regression analysis then discerned the four most available genes with nonzero coefficients to develop a prognostic risk score model to independently predict the OS outcome (Figures 1A,B). Coefficients of four genes (ACADM, ACAT1, CPT1B, and HACD1) were used to calculate the risk score of each sample (Figure 1C).

After removing the samples recorded with a survival time of 0, the fatty acid pathway risk signature was constructed to distinguish the 508 ccRCC samples into high risk and low risk.

**FIGURE 2:**
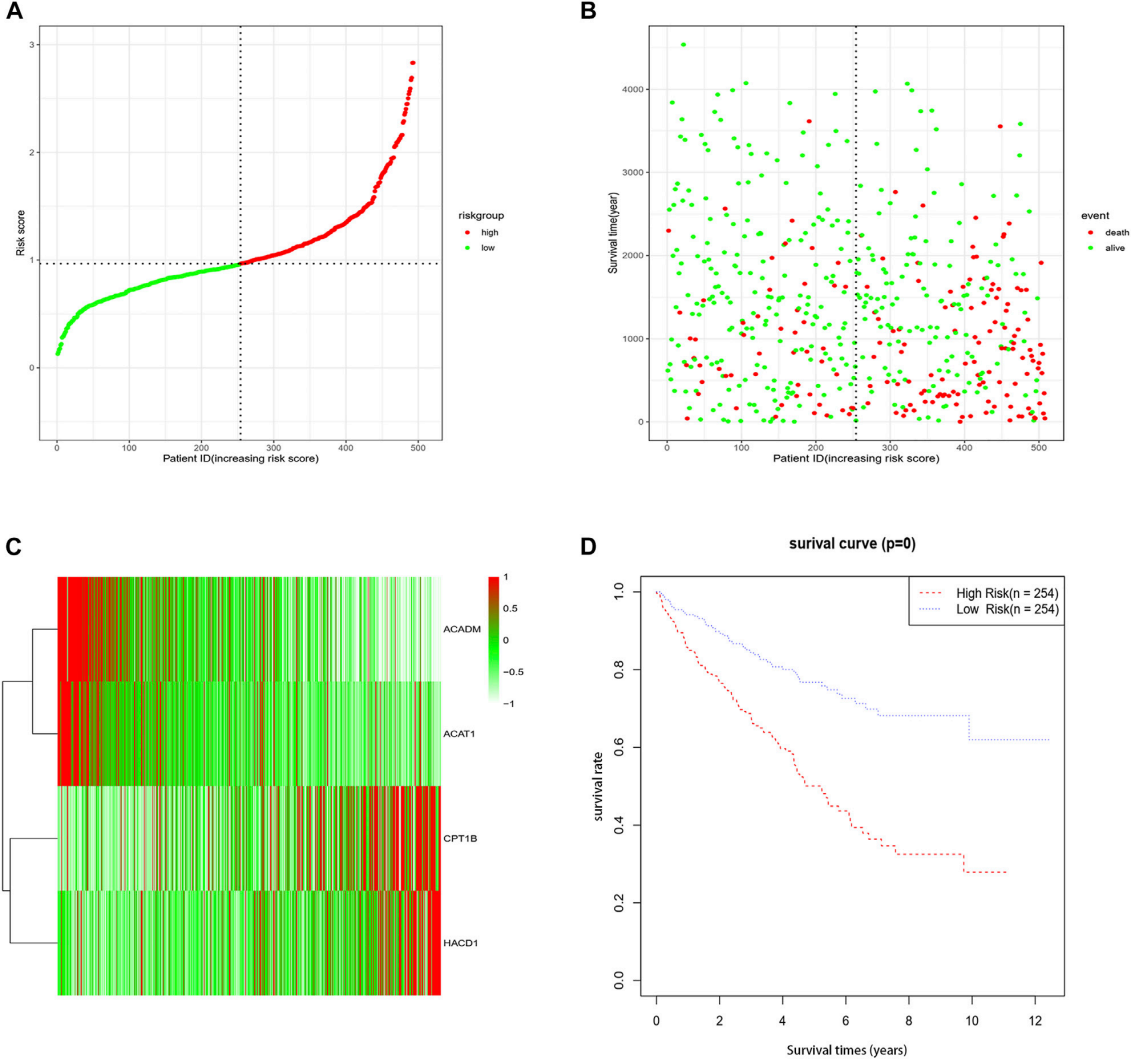
Association between fatty acid metabolism risk signature and patient survival. **(A)** Distribution of each sample by risk scores. **(B)** Association between risk scores and survival time and survival status. **(C)** Heatmap of the expression profile of the four genes. **(D)** K-M survival curve of the high-risk group and low-risk group.

A total of 508 samples were classified as the high-risk group and low-risk group using the median value of the risk score as the cut-off point (Figure 2A). To substantiate the prognostic capability of this risk score model, we plotted the distribution of risk scores and survival times of each sample by risk groups (Figure 2B). As shown in the heatmap of the gene expression profile, the expression of CPT1B and HACD1 was generally higher in the high-risk group than in the low-risk group, while the expression of ACADM and ACAT1 was significantly lower in the high-risk group (Figure 2C). In addition, Kaplan–Meier survival analyses of the high-risk group and low-risk group were performed. Apparently, the OS of the low-risk group was significantly longer than that of the high-risk group (*p* < 0.001) (Figure 2D). As proved above, the risk score model we constructed could predict the prognosis of patients to a certain extent.

### The Functional Enrichment Analysis of High-Risk Group Samples and Low-Risk Group Samples

To further illustrate the fatty acid pathway risk signature, functional enrichment analysis was performed between the high-risk group and the low-risk group. Functional enrichment pathways were analyzed by differentially expressed genes, which were obtained by comparing gene expression between the high-risk group and low-risk group under the condition that |logFC| > 1 and *p* < 0.05.

**FIGURE 3:**
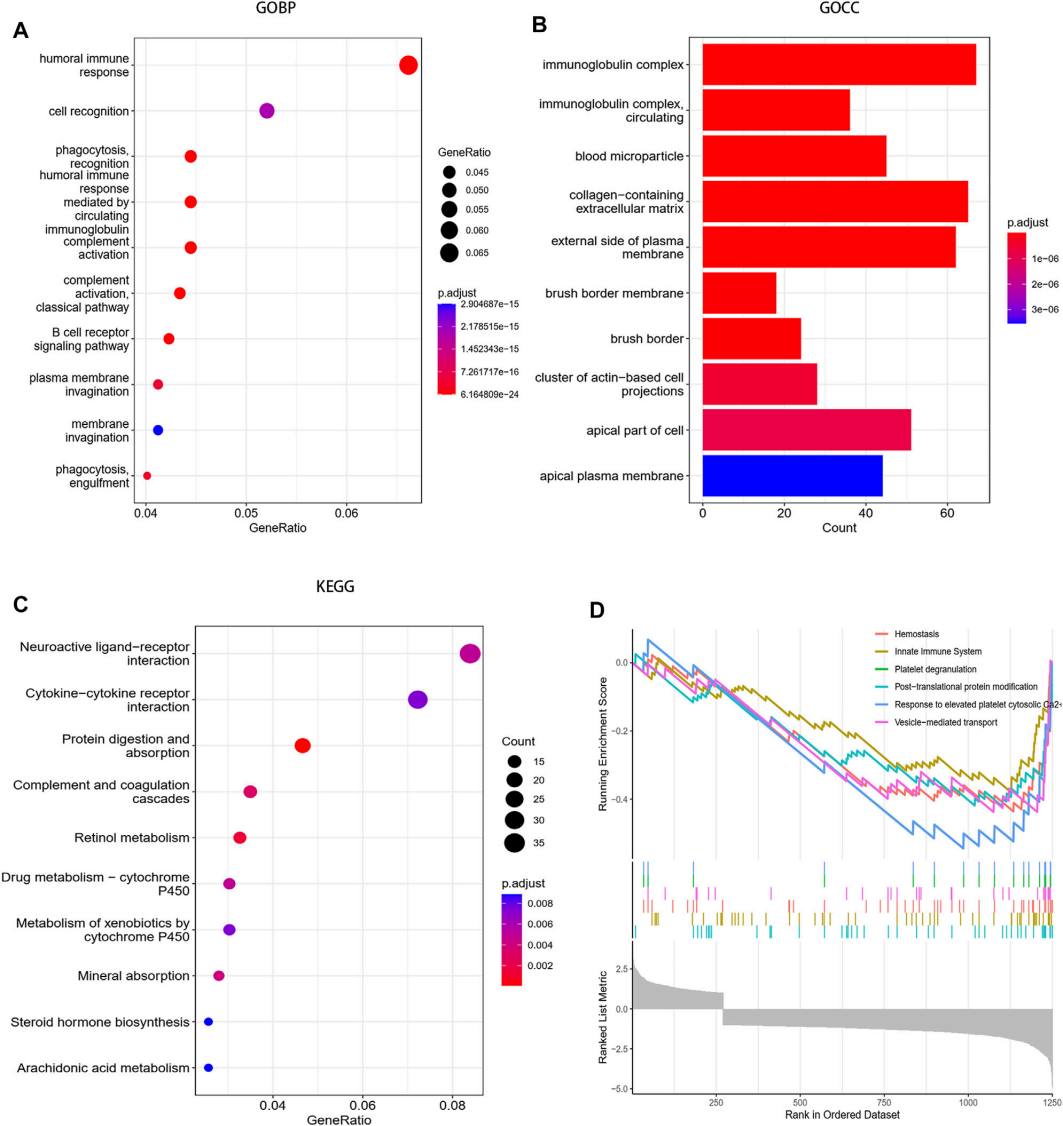
Functional enrichment analysis between the high-risk group and the low-risk group. **(A)** Biological process of GO analysis between the two groups. **(B)** Cellular component of GO analysis between the two groups. **(C)** KEGG analysis between the two groups. **(D)** Reactome pathway analysis between the two groups.

The reactome analysis mainly revealed the involvement of hemostasis and the innate immune system (Figure 3A).

The GO-BP analysis showed that differentially expressed genes were mainly enriched in humoral immune response, cell recognition, phagocytosis recognition, humoral immune response mediated by circulating and immunoglobulin complement activation, etc. Moreover, the GO-CC analysis also revealed the enrichment of cellular components such as immunoglobulin complex, immunoglobulin complex circulating, and blood microparticle (Figures 3B,C).

The KEGG pathway analysis revealed the enrichment of the neuroactive ligand–receptor interaction pathway, cytokine–cytokine receptor interaction pathway, protein digestion and absorption pathway (Figure 3D).

Functional enrichment analysis indicates that differentially expressed genes between the high-risk group and low-risk groups were mainly enriched in immune-related pathways and hemostasis processes in ccRCC.

### The Single-Sample Gene Set Variation Analysis of the TCGA Cohort

**FIGURE 4:**
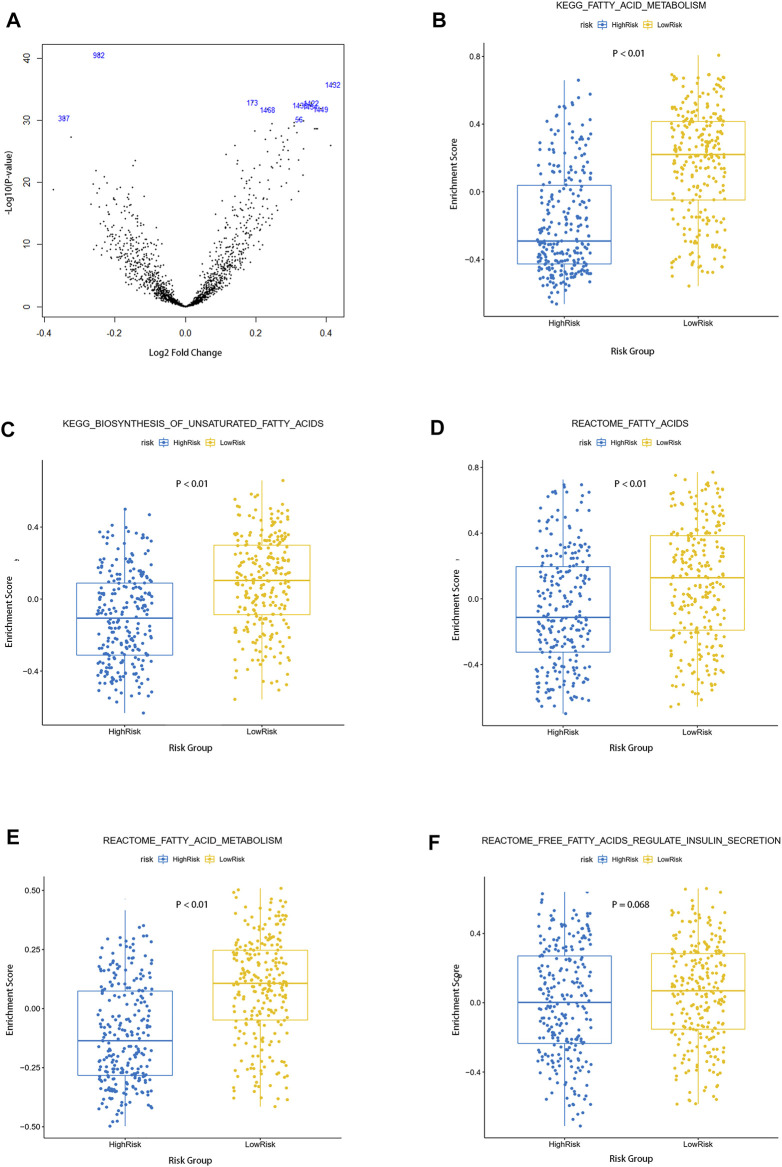
Single sample gene set variation analysis of fatty acid metabolism pathways between the two groups. **(A)** Volcano map of differential pathways between the two groups **(B–F)** Enrichment of five fatty acid metabolism–related pathways in the two groups.

The gene set enrichment score of each sample in the TCGA cohort was calculated by the “GSVA” R package. Differential analysis of pathway enrichment scores between the high-risk group and low-risk group was performed under the condition that |logFC| > 0.5 and FDR <0.05, and the volcano map of the differential pathways was plotted (Figure 4A). We further analyzed five pathways (KEGG–fatty acid metabolism, KEGG–biosynthesis of unsaturated fatty acids, reactome–fatty acids, reactome–fatty acid metabolism, and reactome–free fatty acids regulate insulin secretion) that were closely related to fatty acid metabolism. As shown in the figures, compared with the low-risk group, the enrichment scores of the high-risk group in the four pathways of KEGG–fatty acid metabolism, KEGG–biosynthesis of unsaturated fatty acids, reactome–fatty acids, and reactome–fatty acid metabolism was predominantly higher, and there were no significant differences between the low-risk group and high-risk groups in the reactome–free fatty acids regulate the insulin secretion pathway (Figures 4B–F). The result of the single-sample gene set variation analysis (ssGSVA) elucidated that fatty acid –metabolism–related pathways were more significantly enriched in a high-risk group and suggested poorer clinical outcomes.

### The Relationship Between Risk Score and Clinical Characteristics

**FIGURE 5:**
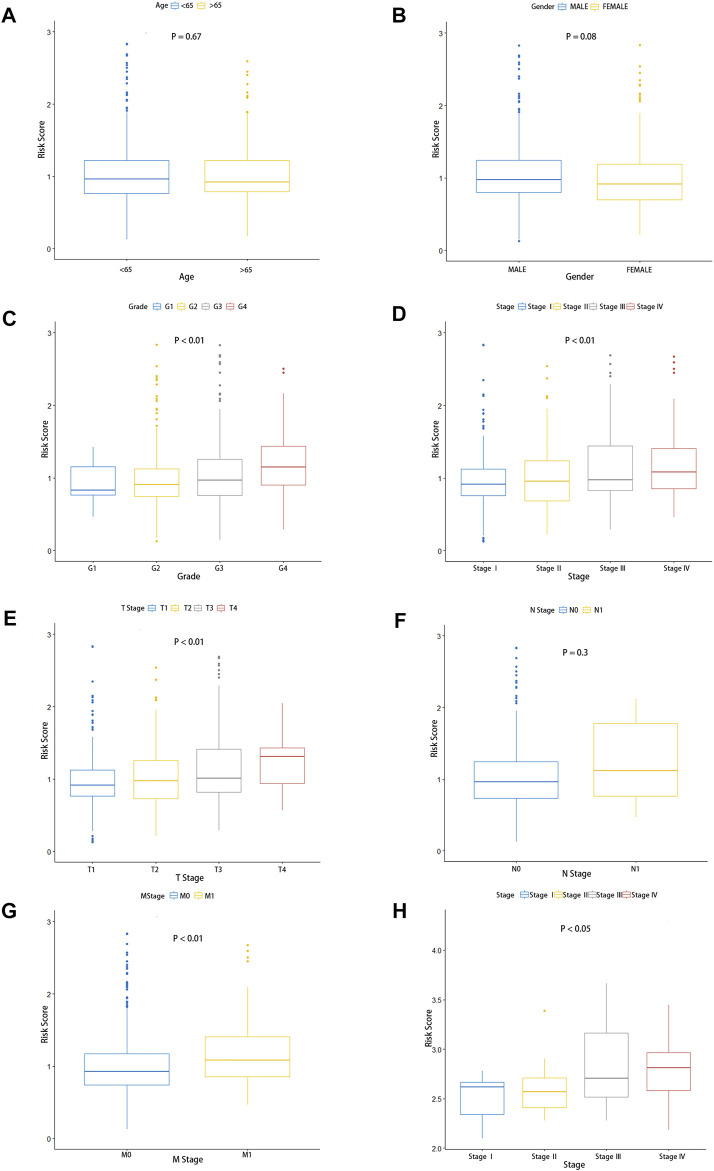
Predictive value of fatty acid metabolism risk signature in the prognosis of patients with ccRCC. **(A–G)** Relationship between risk signature and clinical features, including age **(A)**, gender **(B)**, pathological grade **(C)**, pathological stage **(D)**, tumor size **(E)**, lymphoid invasion **(F)**, and distal metastasis. **(G,H)** Validation of the association between the risk scores and pathological stage in the GEO database.

To investigate the relationship between clinical characteristics and risk scores in patients with ccRCC, we, respectively, studied whether there were significant differences in age, gender, grade, pathological stage, and the AJCC TNM Classification of Malignant Tumors (TNM) stage among different risk groups. Obviously, there were no significant differences in risk score associations with age and gender in ccRCC patients, and we observed that there was no correlation between the risk scores and AJCC T-stages (Figures 5A,B,F). However, the clinical grade (Figure 5C, *p* < 0.001), pathological stage (Figure 5D, *p* < 0.01), AJCC T-stage (Figure 5E, *p* < 0.01), and AJCC M-stage (Figure 5G, *p* < 0.01) were correlated with risk scores, which indicated that the poorer the stage, the higher the risk scores. In addition, the gene expression microarray of 60 ccRCC patients in the dataset GSE150404 was analyzed to further validate the relationship between risk scores and clinical characteristics. We used the same formula to calculate the risk score for each patient, and similarly, the risk score was associated with the pathological stage (Figure 5H), which indicated that the high-risk group had poorer prognosis than the low-risk group. Unfortunately, the clinical information of samples of this dataset contained only pathological stage and no other clinical features and survival status of patients.

Subgroups were classified by age (age >65, age <65), gender (female and male), pathological grade (G1-2, G3-4), pathological stage (STAGEⅠ–Ⅱ; STAGE Ⅲ–Ⅳ), the AJCC T stage (T1-2, T3-4), the AJCC N stage (N0, N1), and the AJCC M stage (M0, M1) in TCGA cohorts. The Kaplan–Meier survival analysis of the high-risk group and low-risk group was performed in each subgroup. As shown in the figures, patients in the low-risk group had a longer overall survival time than those in the high-risk group, except in the subgroup of the AJCC N0 stage (Supplementary Figures S3A–N). These fundings convincingly revealed that the fatty acid metabolism signature could be used to predict the prognosis, survival time, and survival status of ccRCC patients without considering the impact of the clinical features.

### Establishment of the Prognosis Nomogram

**FIGURE 6:**
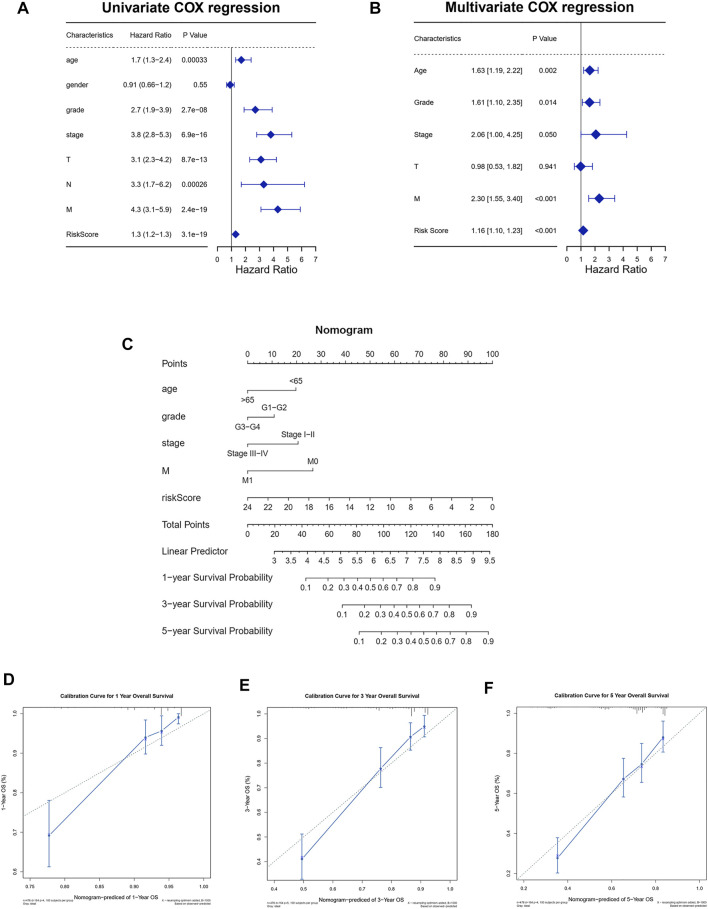
Establishment of the nomogram to predict the survival of patients with ccRCC. **(A)** Univariate Cox regression analysis among risk score and clinical features for survival. **(B)** Multivariate Cox regression analysis based on the univariate Cox regression analysis. **(C)** Establishment of the nomogram predicting survival of patients with ccRCC. **(D–F)** Calibration curves of the nomogram predicting the 1-year **(D)**, 3-year **(E)**, and 5-year **(F)** survival for patients with ccRCC.

From the univariate Cox regression analysis, it could be seen that age, pathological grade, pathological stage, AJCC T stage, AJCC N stage, AJCC M stage, and risk scores were closely related to the prognosis of ccRCC patients. Only one clinical characteristic, gender, did not show a correlation with prognosis in the univariate Cox regression analysis (Figure 6A). Furthermore, as shown in the multivariate Cox regression analysis, age, pathological grade, pathological stage, AJCC T stage, AJCC M stage, and risk score could serve as independent prognostic indicators for ccRCC patients (Figure 6B). We then constructed a nomogram to predict 1-year, 3-year, and 5-year overall survival using these reliable independent prognostic indicators (Figure 6C). The calibration curves at 1-year, 3-year, and 5-year OS validated that the nomogram could achieve the purpose of predicting the overall survival of ccRCC patients (Figures 6D–F).

### Immune-Related Characteristic and Immunotherapy Response Between the High-Risk Group and Low-Risk Group

**FIGURE 7:**
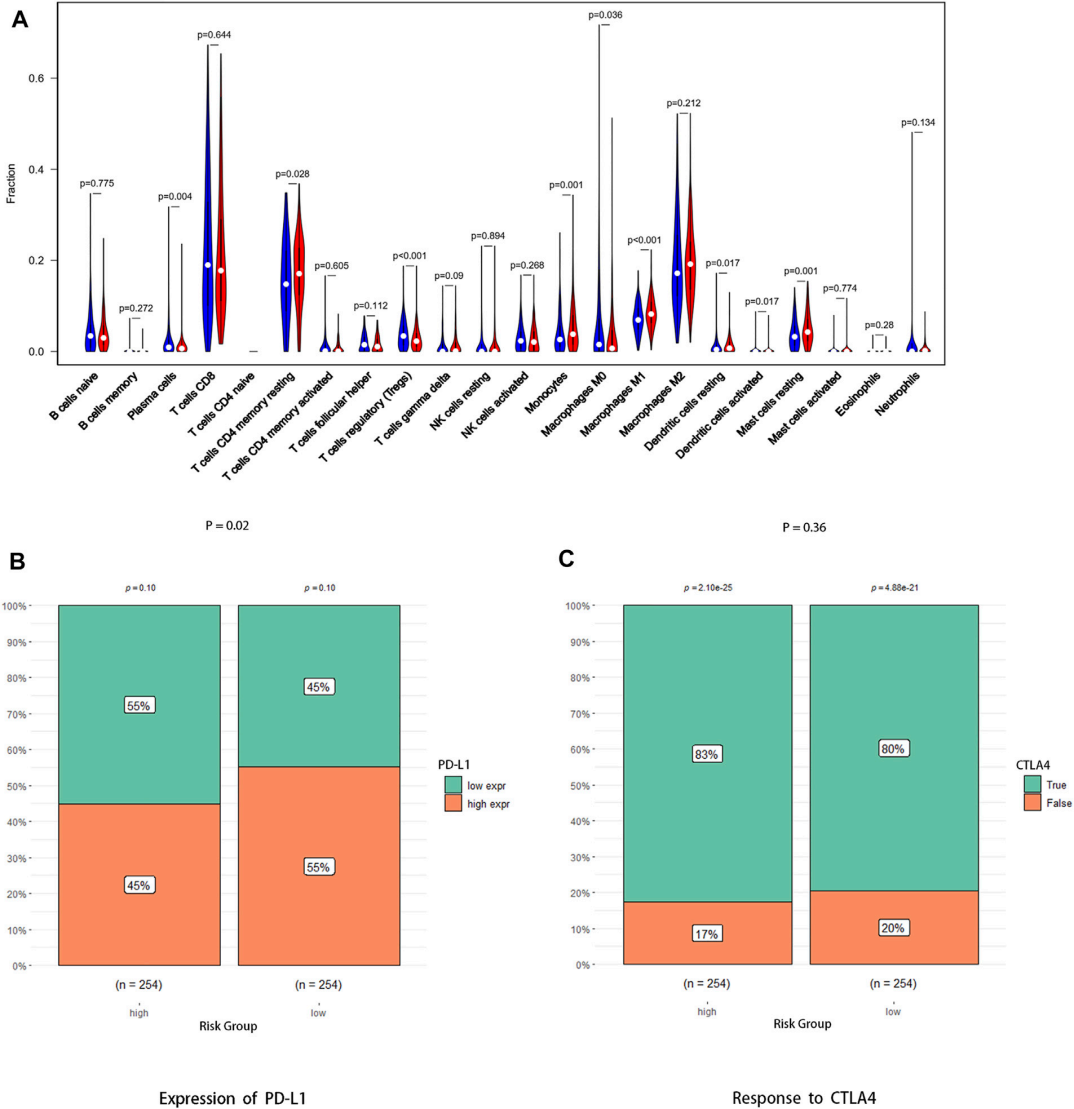
Association between the risk signature and antitumor immune response. **(A)** Abundance ratio matrix of 22 immune cells in the high-risk group and the low-risk group. **(B)** Expression level of PD-L1 between the two groups. **(C)** Response to anti-CTLA4 antibody between the two groups.

The abundance ratio of immune cells for each sample was obtained *via* the “Cell Type Identification by Estimating Relative Subsets of RNA Transcripts (CIBERSORT)” algorithm. As shown in the figures, there were significant differences in immune cell infiltration between the high-risk group and low-risk group, such as memory activated T cells CD4, monocytes, M1 macrophages, and resting mast cells, which were high in the high-risk group. Intriguingly, plasma cells, regulatory T cells (Tregs), and M0 macrophages were more abundant in the low-risk group (Figure 7A, Supplementary Figure S1). In addition, Figure 4B showed that expression of PD-L1 was higher in the low-risk group than in the high-risk group (Figure 7B, *p* < 0.05), which indicates that low-risk group patients may have better efficacy with PD-1/PD-L1 immunotherapy, but there were no significant differences in the expression of CTLA4 between the high-risk group and low-risk group (Figure 7C, *p* = 0.36).

**FIGURE 8:**
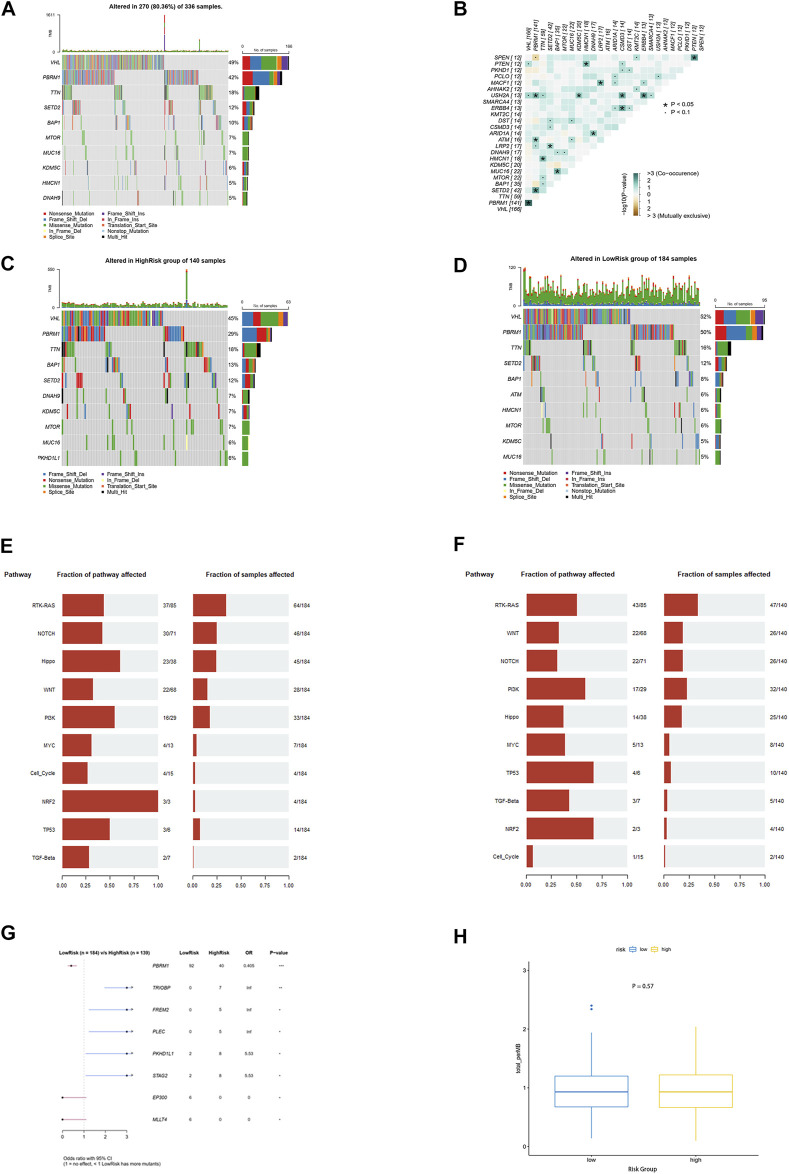
Mutation landscape analysis. **(A)** Top 10 genes with the highest alteration frequency in the TCGA-KIRC cohort. **(B)** Information of mutation co-occurrence. **(C,D)** Top 10 genes with the highest alteration frequency in the high-risk group and the low-risk group. **(E,F)** Top 10 signaling pathways enriched by mutation genes in the high-risk group and the low-risk group. **(G)** Eight mutation genes as independent predictive factors for risk scores **(H)** Tumor mutational burden between the two groups.

The somatic mutation profile of 270 ccRCC samples showed that PBRM1, like VHL, was highly mutated in ccRCC and had a potential co-occurrence with VHL (Figures 8A,B, *p* < 0.05). Moreover, the mutations of PBRM1, EP300, and MLLT4 were more likely to occur in the low-risk group, while the mutation of TRIOBP, FREM2, PLEC, PKHD1L1, and STAG2 was more likely to occur in the high-risk group (Figures 8C,D,G, *p* < 0.05). However, none of these gene mutations was associated with ccRCC prognosis (Supplementary Figures S2A–H) No differences were observed between the two groups in the functional enrichment analysis involved in mutant genes and tumor mutational burden (TMB) (Figures 8E,F,H).

### Response to Targeted Therapy and Correlation With Sunitinib Resistance

**FIGURE 9:**
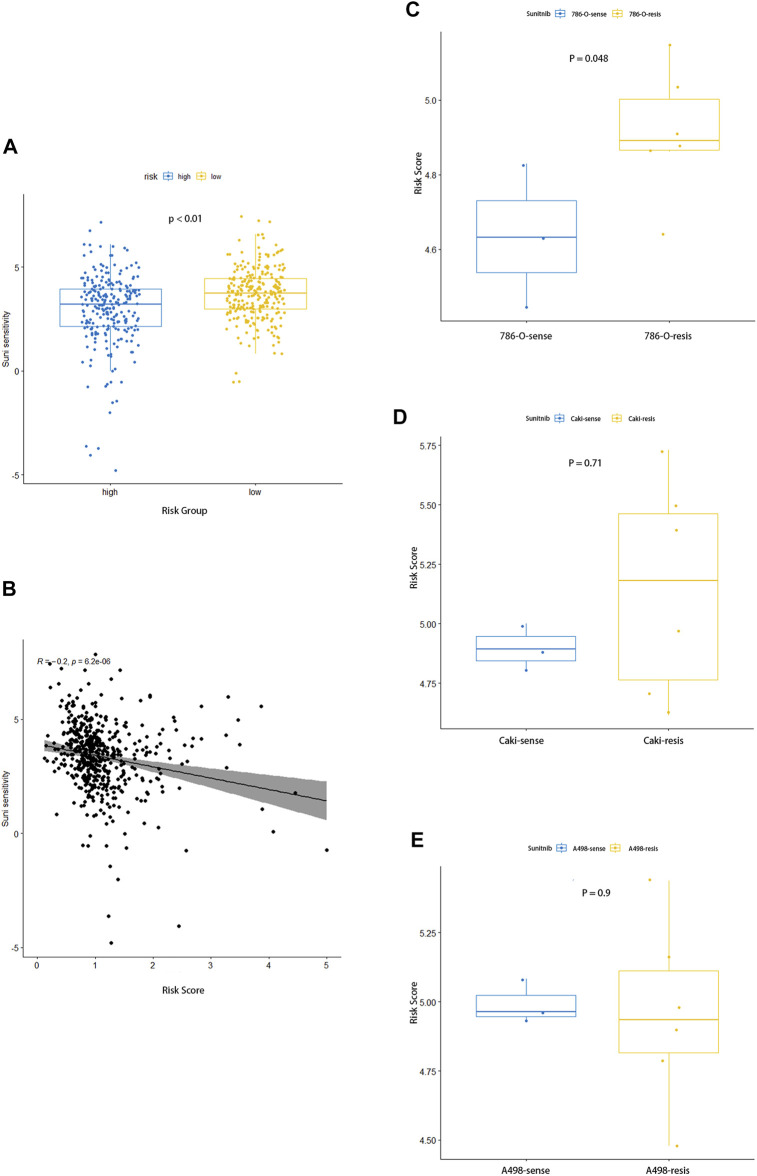
Association between the risk signature and response to sunitinib. **(A)** Comparison of sensitivity to sunitinib between the two groups. **(B)** Correlation analysis for the risk score and the sensitivity to sunitinib. **(C–E)** Distinction in risk score between parental and corresponding resistant renal carcinoma cell lines of 786-O **(C)**, Caki **(D),** and A498 **(E)**.

Fatty acid metabolism–related gene signature has shown a correlation with the prognosis of ccRCC, and we further explore its relationship to the sensitivity of the drug sunitinib. The sensitivity of each sample to sunitinib was calculated by the “pRRophetic” R package, and it can be seen that the high-risk group was significantly less sensitive to the drug than the low-risk group (Figure 9A). Furthermore, the point distribution of risk score and sensitivity value of each sample indicated that risk score was negatively correlated with sensitivity to sunitinib, and its correlation coefficient was −0.2 (Figure 9B, *p* < 0.001). The aforementioned results suggested that the higher risk score not only predicts worse prognosis but also shows desensitization to sunitinib.

The dataset GSE183140 of the GEO database contains three parent renal cell carcinoma cell lines and their corresponding –drug-resistant cell lines, which we used to elucidate the relationship between sunitinib resistance and risk scores. As for Caki-1 and 786-O, the corresponding drug-resistant cell lines had higher risk scores, while no differences were seen in A498, where prominent differences could be seen between 786-O parent and sunitinib-resistant cell lines, and no statistical significance in Caki-1 was observed (Figures 9C–E). As we expected, sunitinib-resistant samples had higher risk scores, which also meant worse prognosis.

### Validation of These Four Genes in Clinical Samples and Cell Lines

**FIGURE 10:**
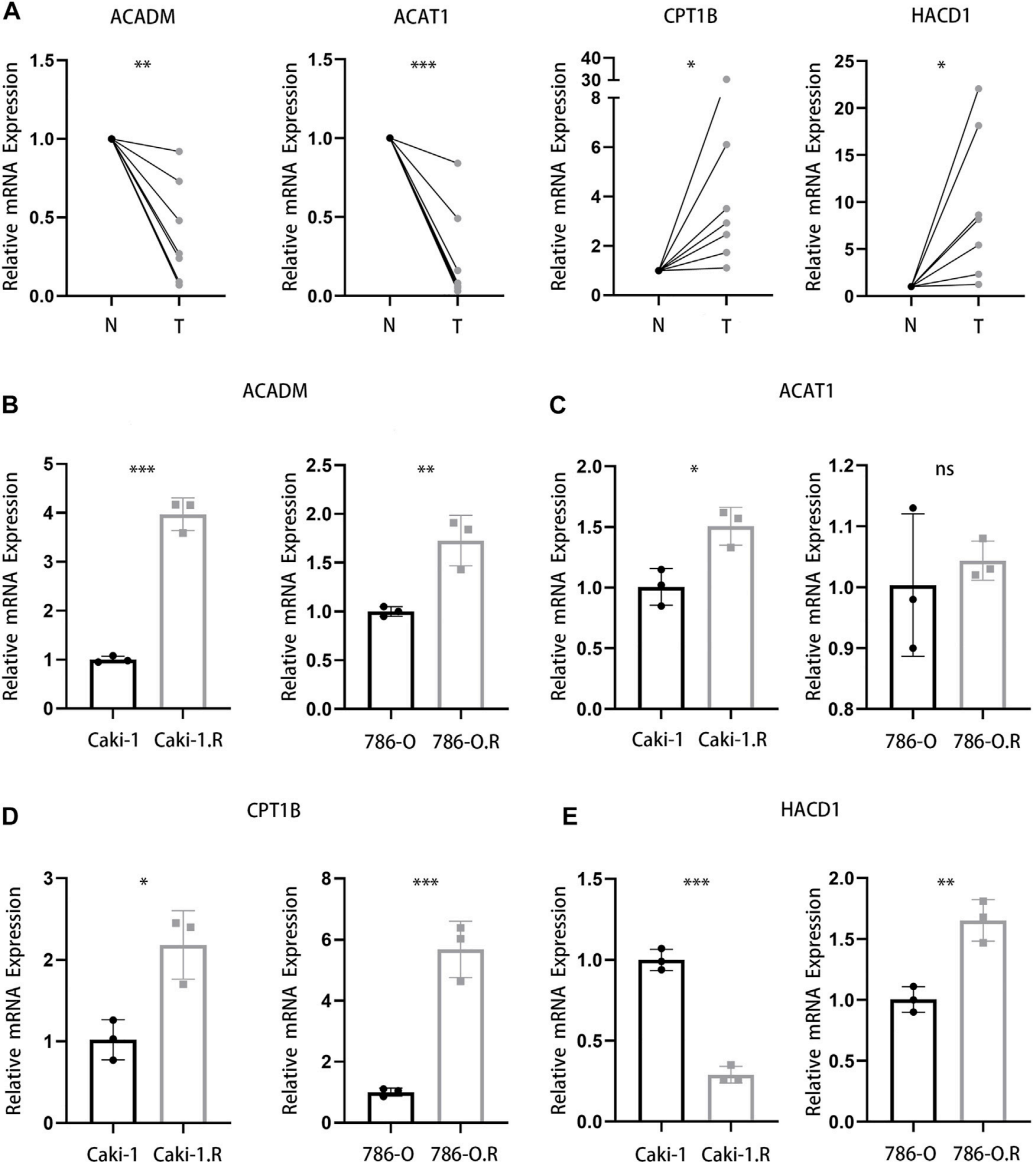
Validation of four genes in clinical samples and cell lines. **(A)** mRNA expression of ACADM, ACAT1, CPT1B, and HACD1 in clinical ccRCC and para-carcinoma samples. **(B–E)** mRNA expression of ACADM **(B)**, ACAT1 **(C)**, CPT1B **(D),** and HACD1 **(E)** in Caki-1, 786-O, and respective sunitinib-resistant cell lines.

To further confirm the results from bioinformatics analysis, the quantitative real time polymerase chain reaction (qRT-PCR) analysis was performed in ccRCC cell lines and clinical samples from ccRCC patients. We detected seven pairs of ccRCC tissues and corresponding para-carcinoma tissues and found a significantly lower level of ACADM and ACAT1 mRNA and a higher level of CPT1B and HACD1 mRNA in tumor tissues (Figure 10A). Then, we studied the mRNA expression of these four genes in ccRCC cell lines (Caki-1 and 786-O) and their respective sunitinib-resistant cell lines. Interestingly, the qRT-PCR analysis of ACADM, ACAT1, and HACD1 showed a different result in ccRCC cell lines and their respective sunitinib-resistant cell lines (Figures 10B,C,E). A significantly higher expression of CPT1B was observed in sunitinib-resistant cell lines than in parental cell lines (Figure 10D), suggesting that CPT1B plays a key role in the mechanism of sunitinib resistance in ccRCC.

## Discussion

ccRCC, as the most common subtype of renal cell carcinoma, tends to show worse prognosis and malignant features, which indicated that the treatment for ccRCC focused mainly on comprehensive treatment (Shuch et al., 2015). Although surgical resection of early localized ccRCC was still a curative method, systemic treatment was dominant for advanced tumors, especially metastatic tumors (Barata and Rini, 2017). Over the past years, medical treatment for ccRCC has transitioned from a nonspecific immune approach to targeted therapy against vascular endothelial growth factor (VEGF) and now to novel immunotherapy agents (Garcia and Rini, 2007). The current systemic treatment of targeted therapies has been shown to improve progression-free survival in metastatic RCC (Bedke et al., 2016). However, some patients were still insensitive to targeted therapy or showed resistance after short-term therapy. Surprisingly, recent studies have demonstrated that the addition of LDL cholesterol increased activation of PI3K/AKT signaling, which coincided with reduced antitumor therapy such as sunitinib against ccRCC (Naito et al., 2017; Makhov et al., 2018). The aforementioned findings all reflected activation of the lipid metabolism pathways in the treatment of ccRCC and the mechanism of drug resistance.

As is known to all, the reprogramming of the cellular metabolism played an essential role in tumor development (Pavlova and Thompson, 2016). Furthermore, kidney cancer, especially clear cell renal cell carcinoma, has been aptly labeled a metabolic disease (Linehan et al., 2010; Linehan and Ricketts, 2013). More and more findings suggested the upregulation of lipid storage and utilization of lipids for membrane synthesis in ccRCC (Gebhard et al., 1987; von Roemeling et al., 2013; Horiguchi et al., 2008). Intriguingly, increased levels of fatty acylcarnitines and carnitine in ccRCC were compared with those of normal controls, and these alterations correlated with kidney cancer grade, which suggested that fatty acid metabolism played an important role in the occurrence, progression, and even treatment of ccRCC (Horiguchi et al., 2008; Wettersten et al., 2017). However, current studies have not systematically elaborated on the specific mechanism of different lipid metabolism, especially fatty acid metabolism pathways, in the occurrence, progression, treatment, and prognosis of ccRCC, and the exploration of fatty acid metabolism pathways in ccRCC can help us better study its significance and provide new therapeutic ideas and strategies.

In this study, considering that fatty acid metabolism pathways are closely related to the progression, treatment, and mechanism of drug resistance of ccRCC, a fatty acid metabolism–related risk signature was established by screening differentially expressed genes in tumor and normal samples in the TCGA cohort using the multivariate Cox regression and LASSO Cox regression analysis, which had the potential to predict prognosis, overall survival, response to targeted agents, and immunotherapy in patients with ccRCC. The high-risk group tended to have a worse prognosis, shorter survival, and lower sensitivity to targeted agents and immunotherapy. This risk model involved four fatty acid metabolism–related genes, including ACADM, ACAT1, CPT1B, and HACD1.

It had been reported that ACADM is associated with the progression of some tumors, especially those closely related to lipid metabolism, such as neuroblastoma and breast cancer (Ma et al., 2021; Hsieh et al., 2019; Yu et al., 2019). Interestingly, we found that ACADM plays an anticancer role in most tumors such as hepatocellular carcinoma and neuroblastoma (Hsieh et al., 2019; Ma et al., 2021). However, it has been reported that ACADM enhances the invasion and metastasis ability of breast cancer cells (Yu et al., 2019). In addition, ACADM has been shown as a potential biomarker for kidney cancer, and consistent with this study, ACADM is downregulated in renal cancer. However, the study did not address the potential impact of ACADM on RCC and its association with clinical features of patients with RCC (Xu et al., 2021). ACAT1 has also been identified as a possible anticancer therapeutic target and is closely involved in lipid metabolism and antitumor immune response in recent studies (Yang et al., 2016; Goudarzi, 2019; Gu et al., 2020). Moreover, it had been reported that ACAT1 is essential for the progression of ccRCC, but these studies only discussed the relationship between ACAT1 and prognosis, ignoring its important role in immunotherapy (Chen et al., 2019). Surprisingly, CPT1B has been shown to be associated with mechanisms of drug resistance in multiple cancers such as bladder cancer, prostate cancer, etc. (Iwamoto et al., 2018; Wang et al., 2018; Vantaku et al., 2019; Abudurexiti et al., 2020) However, there are still no relevant studies on the relationship between CPT1B and drug resistance in ccRCC. Few studies have been carried out on HACD1, and it has only been reported as an independent prognostic factor in uveal melanoma (UVM) (Xu et al., 2019). Therefore, this study is the first to identify these four genes as a prognostic model and find their relationship with antitumor immune response and drug resistance.

The fatty acid metabolism–related risk model can predict 1-, 3-, or 5-year survival of patients with ccRCC by calculating the risk score of patients. By analyzing the risk scores of patients in each clinically characterized subgroup, such as pathological grade, pathological stage, and the AJCC TNM stage, we found that survival differences can be shown between the two groups in each subgroup through this model. The risk model we constructed serves as a biomarker to compare prognosis and survival among patients at the same pathological stage.

Tumors must be achieve the evasion of immune surveillance in order to progress and metastasize (Gajewski et al., 2013). Tumors limit the host immune response *via* upregulation of PD-1 ligand (PD-L1) and its ligation to PD-1 on antigen-specific CD8^+^ T cells, which suggests the expression of PD-L1 in tumor tissues is closely related to the efficacy of PD-L1 blockade (Tumeh et al., 2014). In our study, the low-risk group had a higher expression of PD-L1 in the tumor tissues than in the high-risk group, indicating a better efficacy of PD-L1 blockade, suggesting that the high-risk group could not benefit from PD-L1 blockade and tended to be immunotherapy-resistant. Intriguingly, although no difference was shown between the responses of the two groups to anti-CTLA4 antibodies, both of which had reliable response rates, meaning that anti-CTLA4 antibodies could be actively attempted in the high-risk group. In addition, TMB could also be used as an effective biomarker to predict the response of tumor tissues to PD-L1 blockade (Chan et al., 2019). However, no significant difference in TMB between the two groups was observed in our study. These results indicate that the risk model established by us can predict ccRCC patient response to immunotherapy to a certain extent, but whether there is an association between it and TMB needs to be verified by more research.

A comparison of responses to sunitinib in the high-risk group and low-risk group can further help understand the significance of this risk model in the treatment of ccRCC. It was found that samples with higher risk scores were less sensitive to sunitinib, and there was a negative correlation between them, suggesting that patients in the high-risk group tended to be desensitized to sunitinib treatment. Furthermore, in comparison of three different human renal carcinoma cell lines and their corresponding sunitinib-resistant cell lines, the risk scores of the sunitinib-resistant 786-O cell line were significantly higher than those of the parental cell line, while this trend was not observed in the other two cell lines (Caki-1 and A498).Through verification in ccRCC cell lines and their respective sunitinib-resistant cell lines, it was found that CPT1B played a more important role in the drug-resistance mechanism of ccRCC. Therefore, this risk model is also helpful in predicting patient response to targeted agents such as sunitinib and their tendency to develop resistance, which shows that the model has the potential to guide patient treatment. Unfortunately, the distinctness between different cell lines suggests that this ability may not cover all patients with ccRCC and its effectiveness deserves more research.

In summary, the fatty acid metabolism–related risk model can be used to predict survival, response to immunotherapy as well as targeted therapy, and propensity to drug resistance in patients with ccRCC. Risk scores can be associated with numerous clinical features, such as pathological grade, pathological stage, AJCC T stage, and AJCC M stage. Therefore, this risk model can not only serve as a biomarker to assess the prognosis of patients but also achieve personalized treatment, which means an optimal treatment approach. However, there are still limitations to our study. First, distinctness can be seen in response to targeted drugs between the different renal cell lines, and the mechanisms behind this need to be further studied. Second, we found that the hemostasis process also differed significantly between the two groups in the functional enrichment analysis, but we did not further investigate its potential mechanisms. Last, the functional and molecular mechanisms of these four genes for the progression of ccRCC need to be investigated in the next stage, and the mechanism of the differences in response to therapies remains unclear.

## Data Availability

Publicly available datasets were analyzed in this study. These data can be found here: TCGA-KIRC-RNA-Seq: https://portal.gdc.cancer.gov GSE183140 and GSE150404: https://www.ncbi.nlm.nih.gov/geo/.

## Abbreviations

ccRCC, clear cell renal cell carcinoma; DEGs, differentially expressed genes; GO, Gene Ontology; KEGG, Kyoto Encyclopedia of Genes and Genomes; GSVA, the Gene Set Variation Analysis; ssGSVA, the single-sample Gene Set Variation Analysis; TMB, Tumor Mutation Burden; TIDE, Tumor Immune Dysfunction and Exclusion; OS, overall survival.
