# Supplementary material for: A Fatty Acid Metabolism Signature Associated With Clinical Therapy in Clear Cell Renal Cell Carcinoma
Source: Front Genet. 2022 Jul 8;13:894736. doi: 10.3389/fgene.2022.894736 (PMC9304894; doi:10.3389/fgene.2022.894736)
Supplement: Supplementary file 1 [file DataSheet4.DOCX]

**Supplemental information**

**
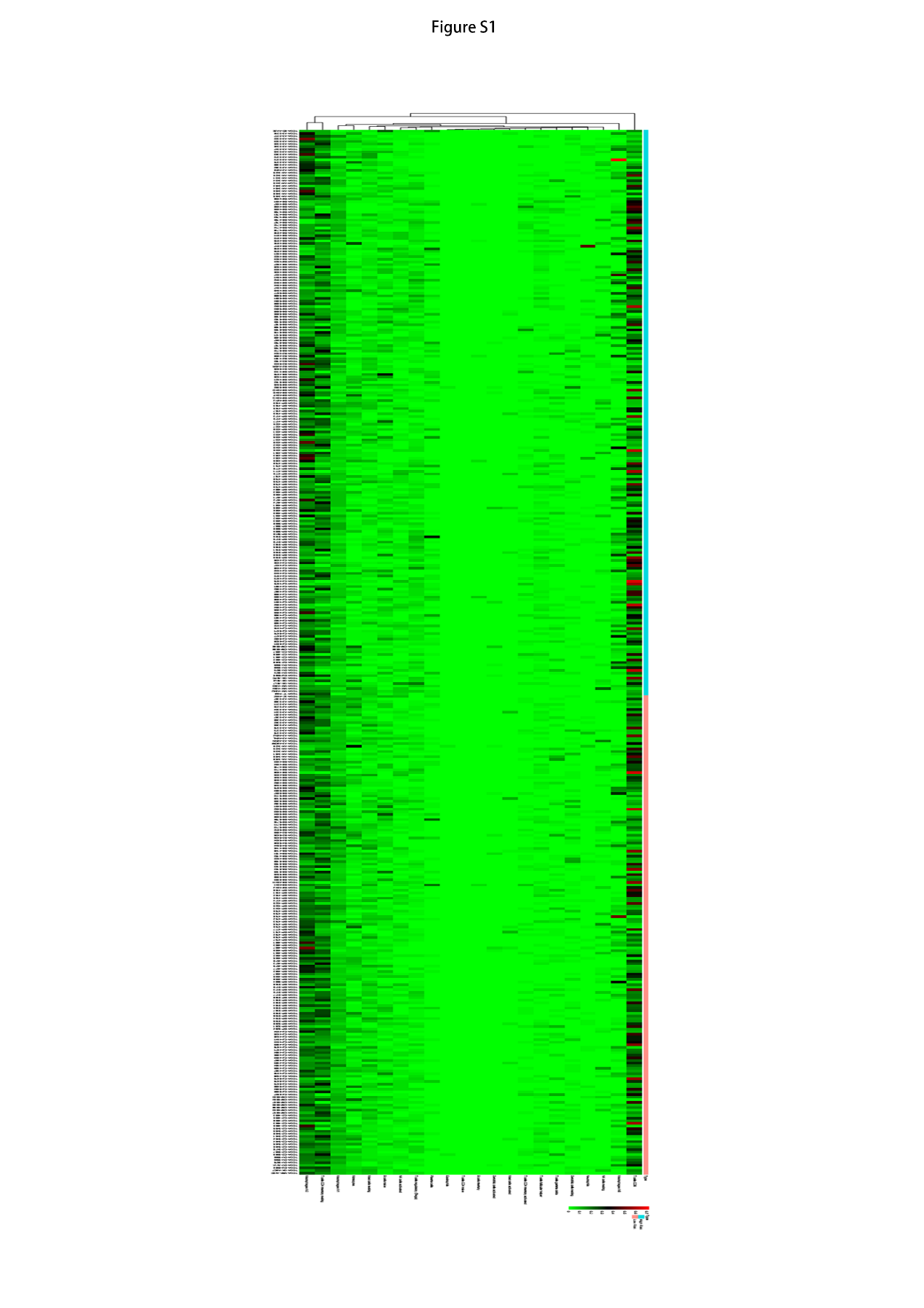
**

**Figure S1 Heat map comparing the abundance ratio of immune cell infiltration between the high-risk group and low-risk group.**


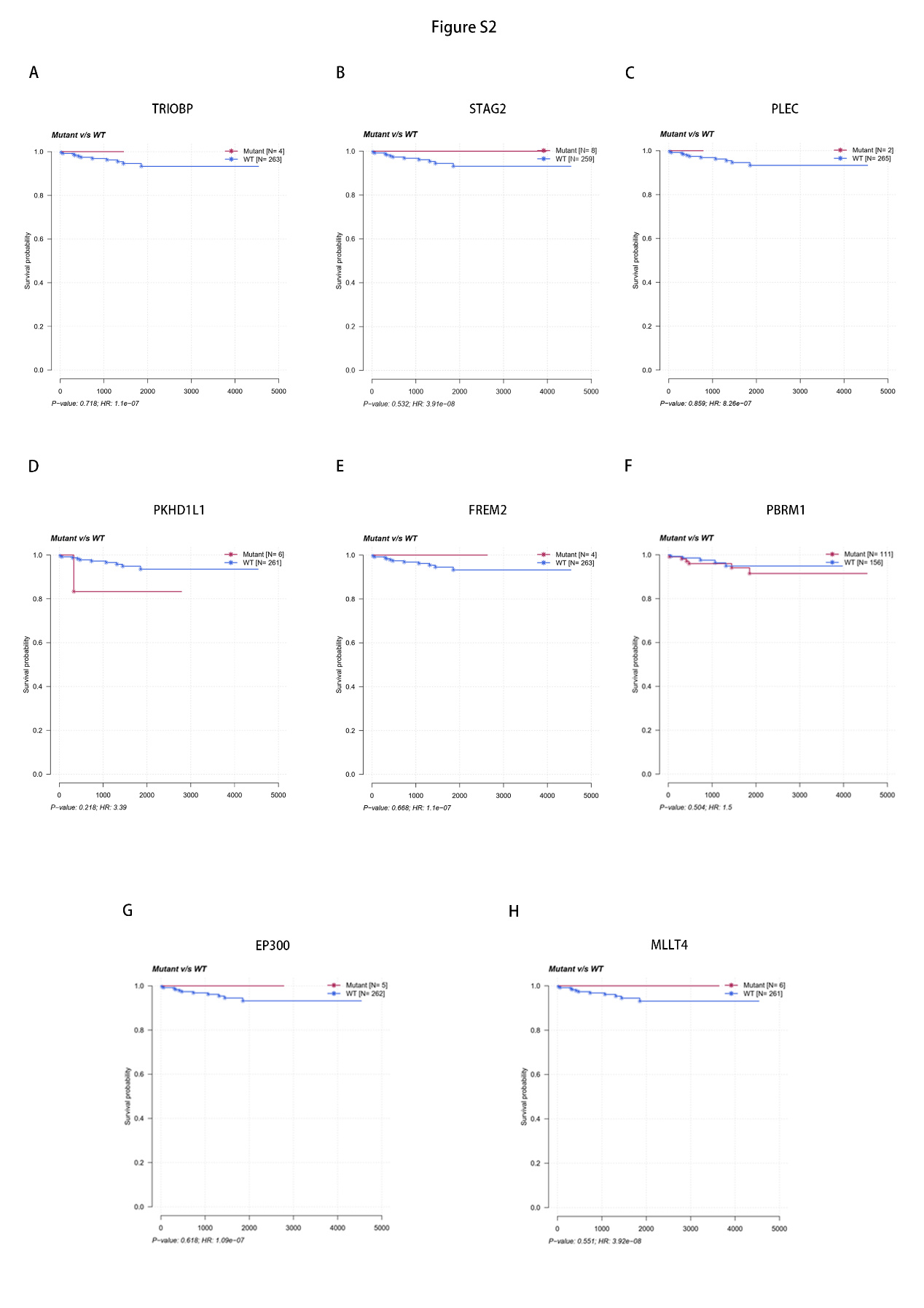


**Figure S2 Survival analysis of 8 mutant genes and corresponding wild type genes.**


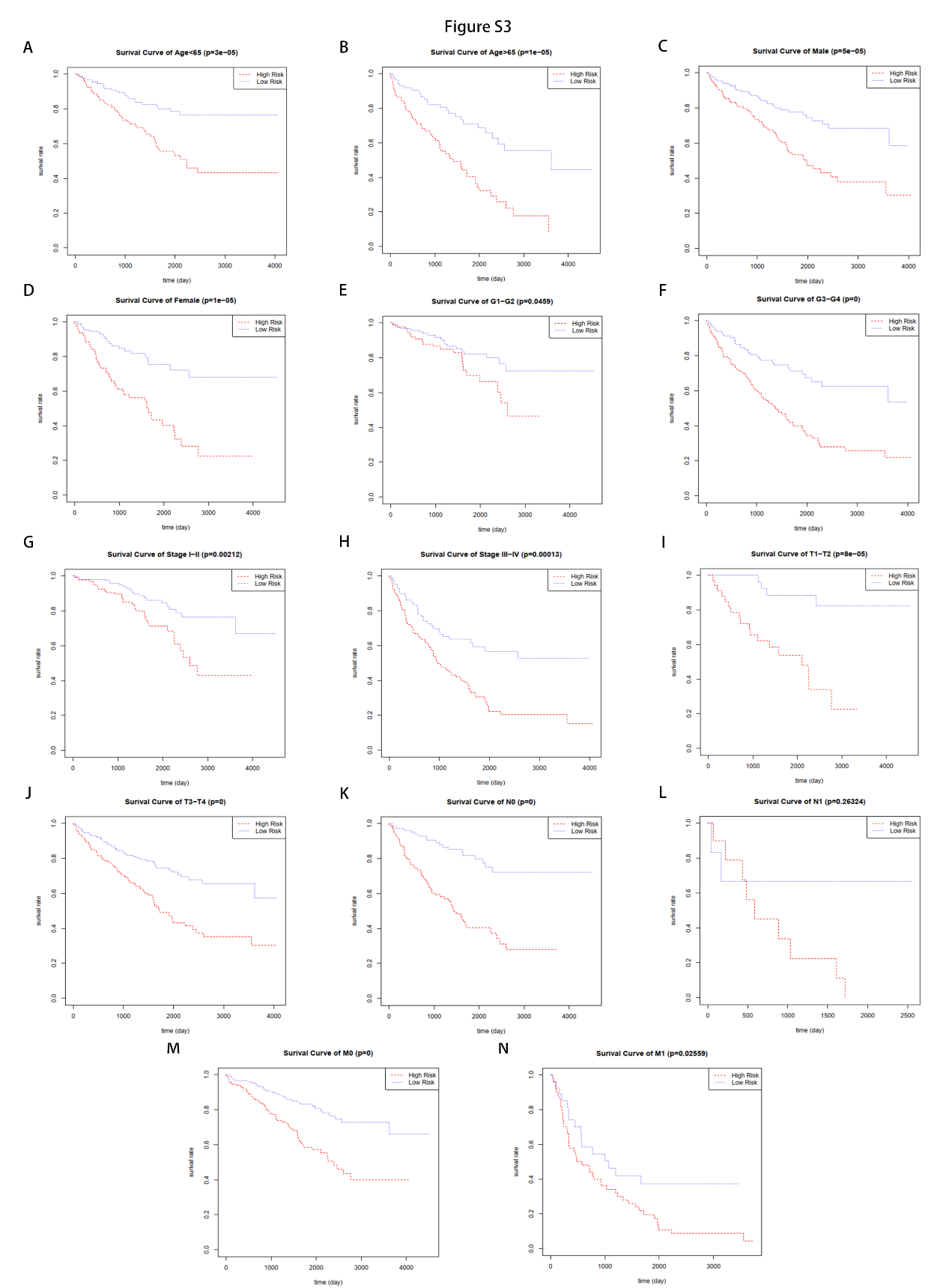
**Figure S3** **Survival analysis of subgroups of individual clinical features.**

(A-N) Survival analysis for the high-risk group and the low-risk group stratified by age(A-B), gender(C-D), pathological grade(E-F), pathological stage(G-H), tumor size(I-J), lymphoid invasion(K-L), distal metastasis(M-N).
