# Supplementary figures and images for: A Fatty Acid Metabolism Signature Associated With Clinical Therapy in Clear Cell Renal Cell Carcinoma
Source: Front Genet. 2022 Jul 8;13:894736. doi: 10.3389/fgene.2022.894736 (PMC9304894; doi:10.3389/fgene.2022.894736)

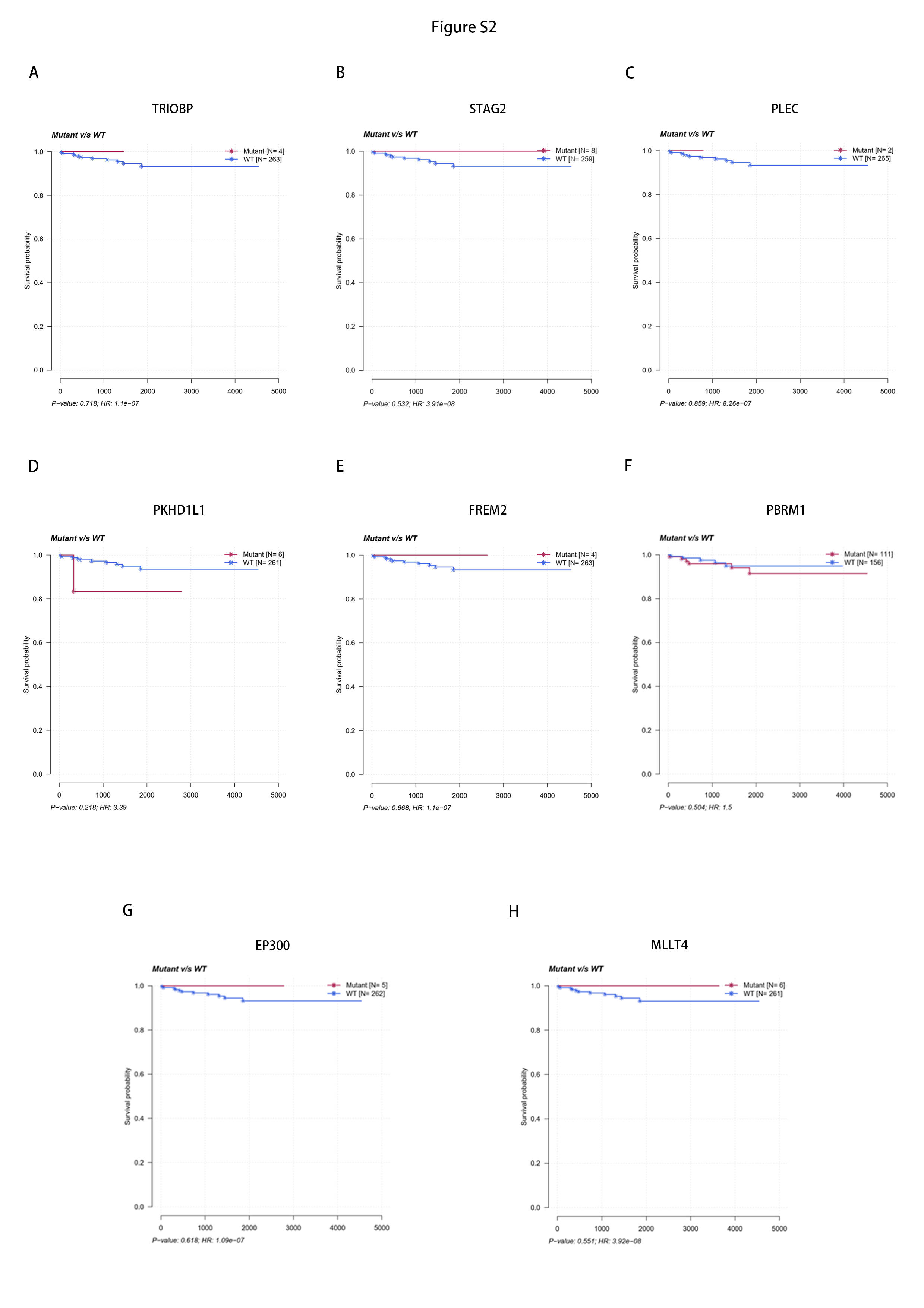

Supplement: Supplementary file 3 [file Image3.TIF]

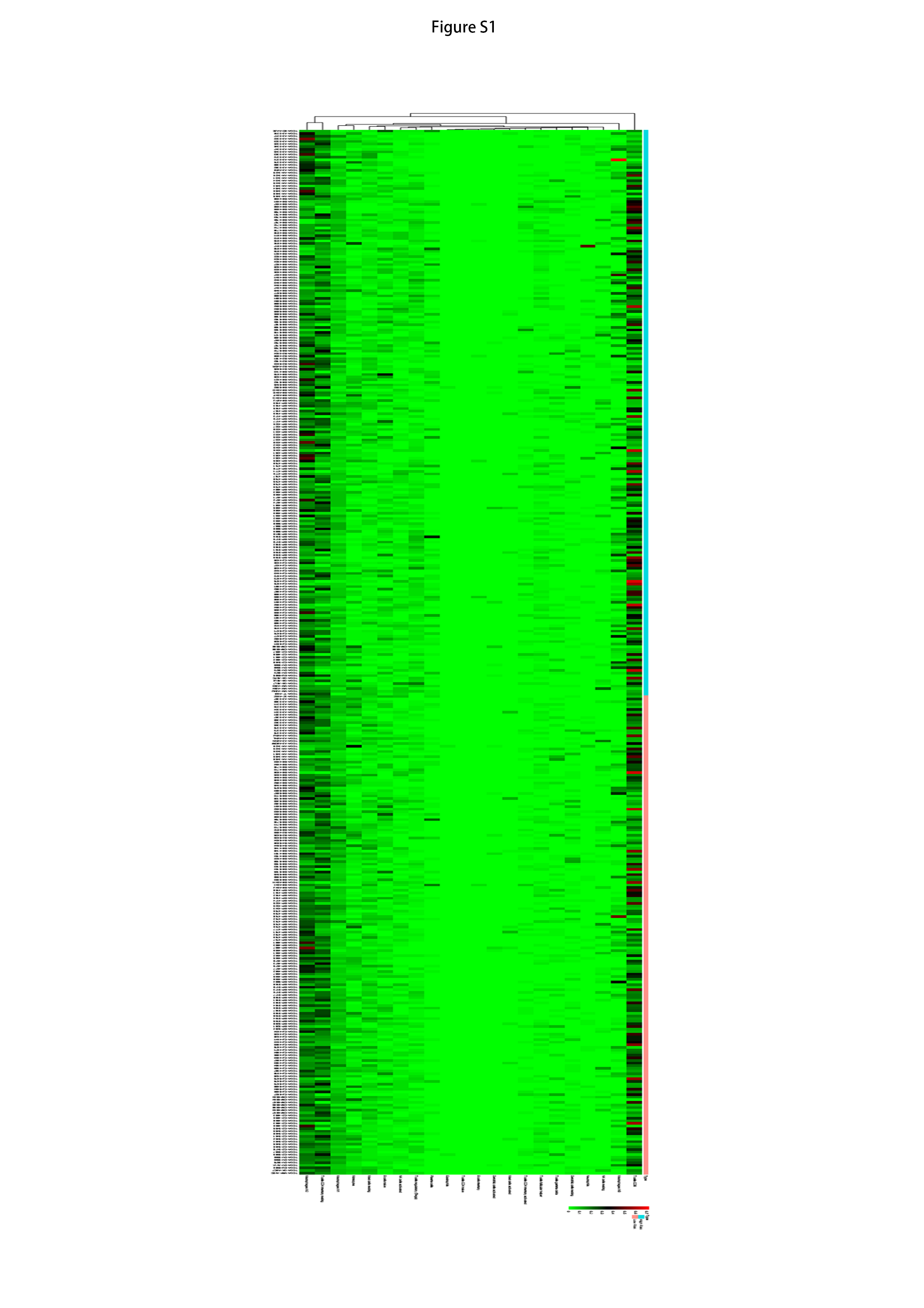

Supplement: Supplementary file 4 [file Image2.TIF]

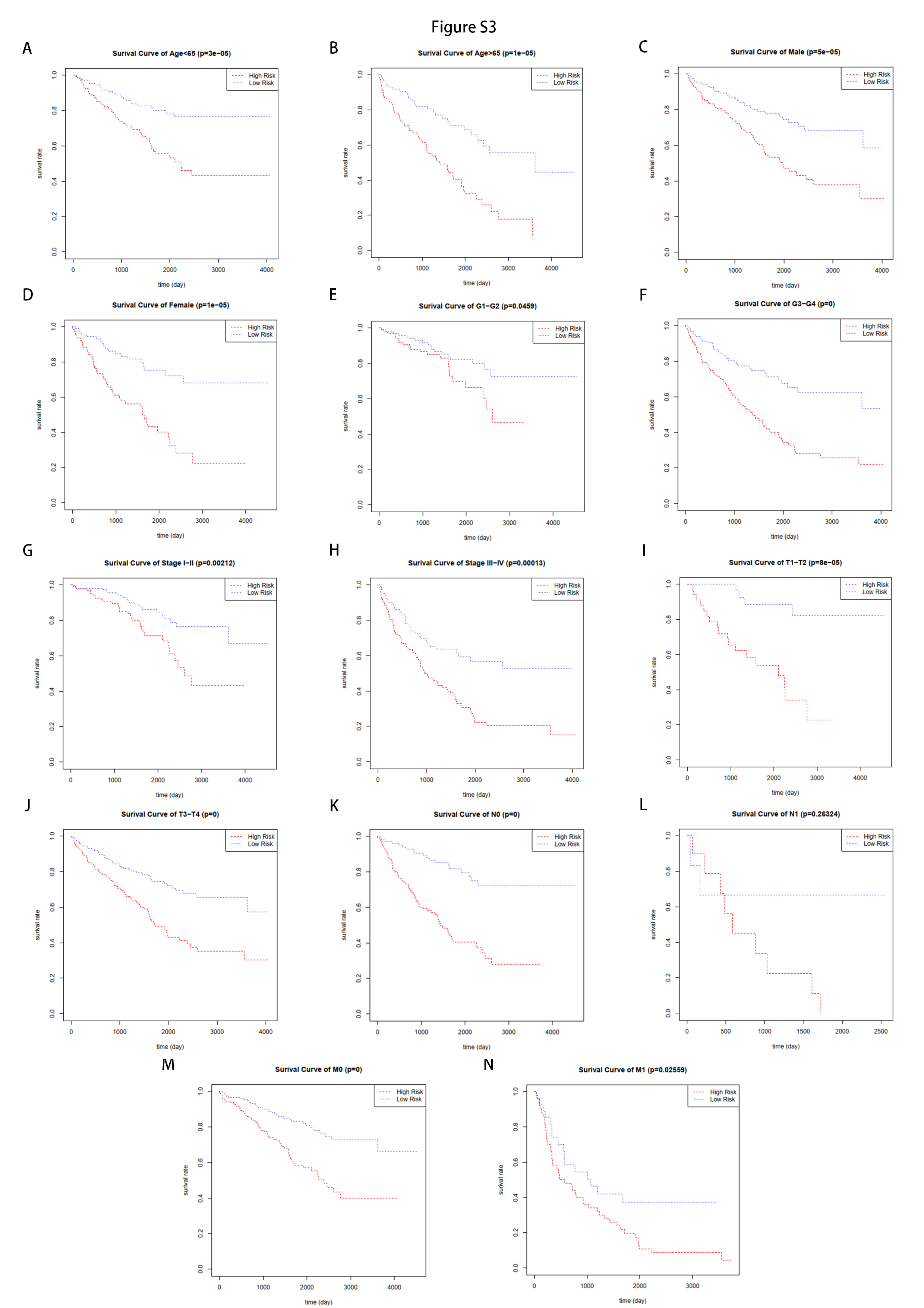

Supplement: Supplementary file 6 [file Image1.TIF]
